# Rapid identification of *Staphylococcus aureus* based on a fluorescence imaging/detection platform that combines loop mediated isothermal amplification assay and the smartphone-based system

**DOI:** 10.1038/s41598-022-25190-6

**Published:** 2022-11-30

**Authors:** Patricia Cabrales-Arellano, Edward Park, Martha Minor, Efren Delgado, Delia Valles-Rosales, Heidi Taboada, José Espiritu, Jianzhong Su, Young Ho Park

**Affiliations:** 1 Family and Consumer Science Department, New Mexico State University, Las Cruces, NM 88003 USA; 2 Biology Department, New Mexico State University, Las Cruces, NM 88003 USA; 3 Industrial Management and Technology Department, Texas A&M University Kingsville, Kingsville, TX 78363 USA; 4 Mechanical and Industrial Engineering Department, Texas A&M University Kingsville, Kingsville, TX 78363 USA; 5 Department of Mathematics, University of Texas at Arlington, Arlington, TX 76019 USA; 6 Mechanical Engineering Department, New Mexico State University, Las Cruces, NM 88003 USA

**Keywords:** Assay systems, Biological fluorescence

## Abstract

Food associated diseases pose significant public health threat in the United States. Health risks associated with food-borne pathogens drive the need for constant monitoring of food products. An efficient method that can diagnose food-borne pathogens rapidly will be invaluable and in high demand. In this study, we showed the feasibility of a novel rapid detection platform based on fluorescence imaging/detection that combines a user-friendly, portable loop mediated isothermal amplification (LAMP) reaction device and a smartphone-based detection system. The proposed platform was used to detect *Staphylococcus aureus* which is one of the most important food-borne pathogen especially dairy products. The complete protocol is quicker; the reaction is performed under isothermal conditions and completed in 1 h or less. Experimental results show that LAMP assays were ten-fold more sensitive than PCR-based detection. The proposed smartphone detection system was able to detect and quantify LAMP assay samples containing three different concentrations of *S*. *aureus* from 10^9^ CFU/mL down to 10^3^ CFU/mL. The present proof-of-concept study demonstrated that this platform offers a portable, easy to use method for measuring target pathogens with LAMP amplification.

## Introduction

Food-borne pathogens are defined as biological agents that cause food-borne illness; they can be either viruses, bacteria and eukaryotes like funguses, protozoa or helminths^1^. Every year, the Food and Drug Administration (FDA) reports the outbreaks associated with food-borne pathogens; for instance, in 2020, it reported the contamination of *Escherichia coli*, *Listeria monocytogenes* and *Cyclospora* in clover sprouts, Enoki mushrooms and bagged salad, respectively (FDA 2020). Health risks associated with food-borne pathogens drive the need for constant monitoring of food products. For microbial pathogen detection, the conventional method is bacterial growth on a culture medium then on selective agar; it sometimes involves biochemical or serological testing. This process has some disadvantages because it is time consuming, laborious and detection of non-culturable pathogens is not possible^2^. By following the conventional methodology, the detection of *E. coli* O157 takes around 3 days, *Salmonella* 4–6 days and *Vibrio parahaemolyticus* around 7 days^3^. For this reason, new techniques emerged which provide faster, more reliable and sensitive results; some of the most widely used are the polymerase chain reaction (PCR) and loop mediated isothermal amplification (LAMP). In PCR, the amplification process involves the repeating of three temperature steps. The first step corresponds to DNA double-strand denaturation, the second step involves the primers hybridization and the last one is the DNA extension^4^. After 35–40 cycles, it is possible to obtain thousands to millions of copies starting with a single copy of DNA as a template^5^.

Diverse pathogens in food have been identified by PCR; among them are *E. coli*^6^, *Salmonella*^7^, *V. parahaemolyticus*^8^, *C. perfringens*^9^, and *L. monocytogenes*^10^. Nevertheless, to carry out the PCR, a PCR machine is necessary, and the product needs to be loaded on agarose gel, stained with dye and visualized under UV light^11^. These limitations make PCR a difficult technique to be used in the field.

On the other hand, the LAMP technique developed by Notomi et al.^12^ amplifies DNA through the use of four primers which recognize six distinct sequences in the target DNA. The complete protocol is quicker; the reaction is performed under isothermal conditions and completed in 1 h or less^13^. Food-borne pathogens identified by this technique are *Salmonella*^14^, *S. aureus*^15^, *Campylobacter*^16^, and *L. monocytogenes*^17^. In this study, we demonstrated that the LAMP reaction has great sensitivity for detecting *S. aureus* samples with various concentrations. The LAMP reaction can be analyzed by agarose gel electrophoresis or other detection methods. However, these post-amplification detection techniques require opening of the reaction tubes, which increases the risk of sample contamination. To reduce this risk, products of LAMP amplification can be visualized through fluorescence signal by adding diverse dyes such as SYBR green, calcein, SYTO 9 or berberine^18–20^. Some dyes enable the identification of a positive or negative sample with the naked eye through color change. For example, the use of gold nano-particles in LAMP generates a color change from transparent to pink on positive reactions^21^. On the other hand, the use of hydroxy naphtol blue (HNB) shows a purple coloration in negative reactions compared with a blue coloration in positive ones^19^. Another detection method is analyzing the turbidity derived from magnesium pyrophosphate precipitation which is the by-product of LAMP reactions^22^. In this study, we showed that the application of dyes such as HNB which can be seen with the naked eye and SYBR Safe dye which only needs a blue light to be seen, is an easy way to visually distinguish between positive and negative results.

The evaluation of color change, however, will be dependent on the users’ eye sensitivity to color. For this reason, there is a necessity to develop a device which will help to quantitatively interpret the result in a fast and accurate way and also reduce issues such as false positives. In this study, we developed a novel rapid detection platform that combines a user-friendly, portable LAMP reaction device and a smartphone-based detection system, which enables point-of-cared (POC) testing and diagnostics. This cost-efficient device controls a low-powered isothermal heating module and a LED excitation module via an Arduino microcontroller. The device contains a reaction cuvette to carry out LAMP reaction in the isothermal heater and a USB camera to take an image of the reagent inside the cuvette which is illuminated by an LED. The captured image is processed by a smartphone application developed specifically for this study.

## Materials and methods

### Biological material and DNA extraction

*Staphylococcus aureus* and *Serratia marcescens* strains were grown in Luria–Bertani broth at 30 °C and shaken for 24 h. MRS broth was employed to generate the *Lactobacillus casei* biomass at 35 °C with agitation by 24 h. The cells were then centrifuged at 5000×*g* for 10 min. The supernatant was discarded, and the pellet was used to do the DNA extraction. To carry out this objective, the GeneJet Genomic DNA Purification kit (Thermo Scientific) was used, and the provider instructions were thoroughly followed. DNA quality was evaluated through agarose gel electrophoresis and quantified with Nanodrop 2000 (Thermo Scientific). Furthermore, defatted milk was inoculated with *S. aureus* at different concentrations; samples were boiled for 10 min and centrifuged at 10,000×*g.* The supernatant was stored at 4 °C until its use as a DNA template in the LAMP reaction.

### Primers design and LAMP amplification

To detect *S. aureus,* oligonucleotides were designed based on the FemA gene (DQ352463.1). The primers design was done by Primer Explorer V5 software (http://primerexplorer.jp/lampv5e/index.html). The primer set targeted six regions on the gene via a forward inner primer (FIP), a backward inner primer (BIP), two outer primers (F3 and B3). The best set of primers were FIP 5′CAAAGCCATCATTCTCACGGGTA-ACTTTGTACAAAATCCATCATTGG3′, BIP 5′ACGACAATAACAAAGTAATTGCAGC-AACATAACTTCCCATAGTAGGA3′, F3 5′TTAACTGTTACCGAATTTGACA3′ and B3 5′CCATTACTGGACCACGATTC3′. The oligonucleotides were synthesized by Eurofins Genomics. A typical LAMP reaction consisted of 1 × WarmStart Master Mix (Biolabs), 1.6 μM of FIP and BIP and 0.2 μM of F3 and B3 oligonucleotides. Finally, several DNA and CFU concentrations were used as a template in the final volume reaction of 25 μL. The mix was incubated in a water bath at 65 °C for 45 min and then stopped at 90 °C for 10 min. For visual detection we used both 120 μM of HNB and 1 μL of SYBR Safe dye (Invitrogen) which was previously diluted 1:10. SYBR safe dye was included during the incubation of samples to prevent any contamination and to ensure quantification in one step with the apparatus. LAMP products were visualized through agarose gel under a blue light transilluminator and with the naked eye.

### Design of a portable device for LAMP reaction

**Figure 1:**
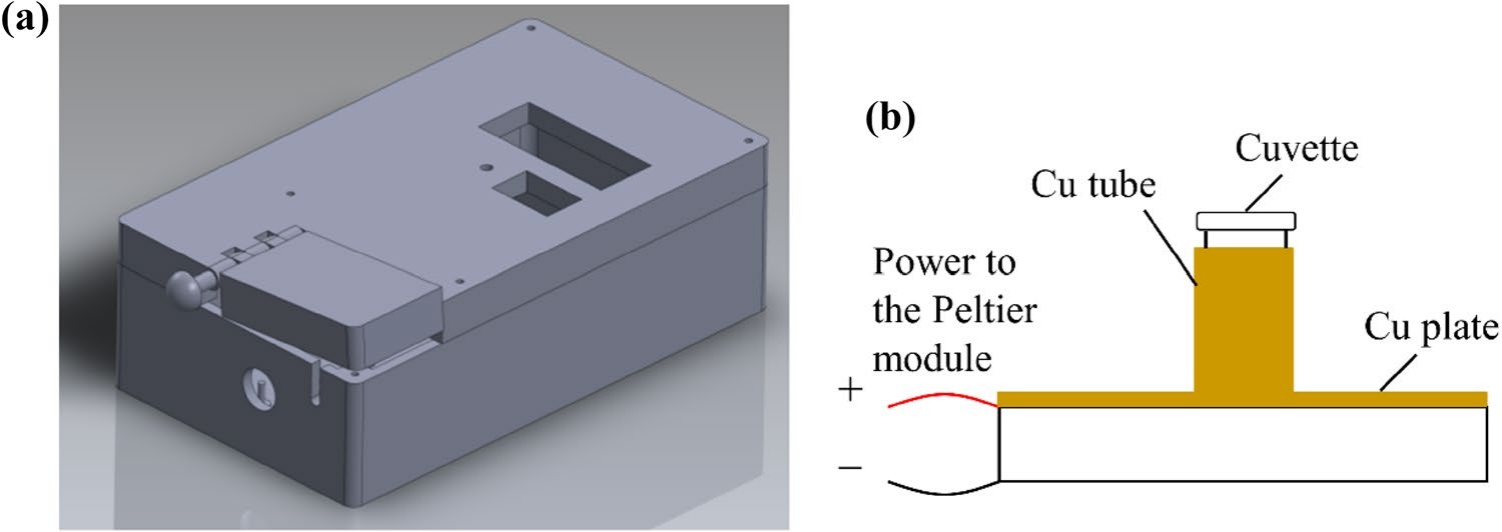
(**a**) 3D design of portable LAMP detection device; (**b**) schematic of cuvette encased by Peltier module.

**Figure 2:**
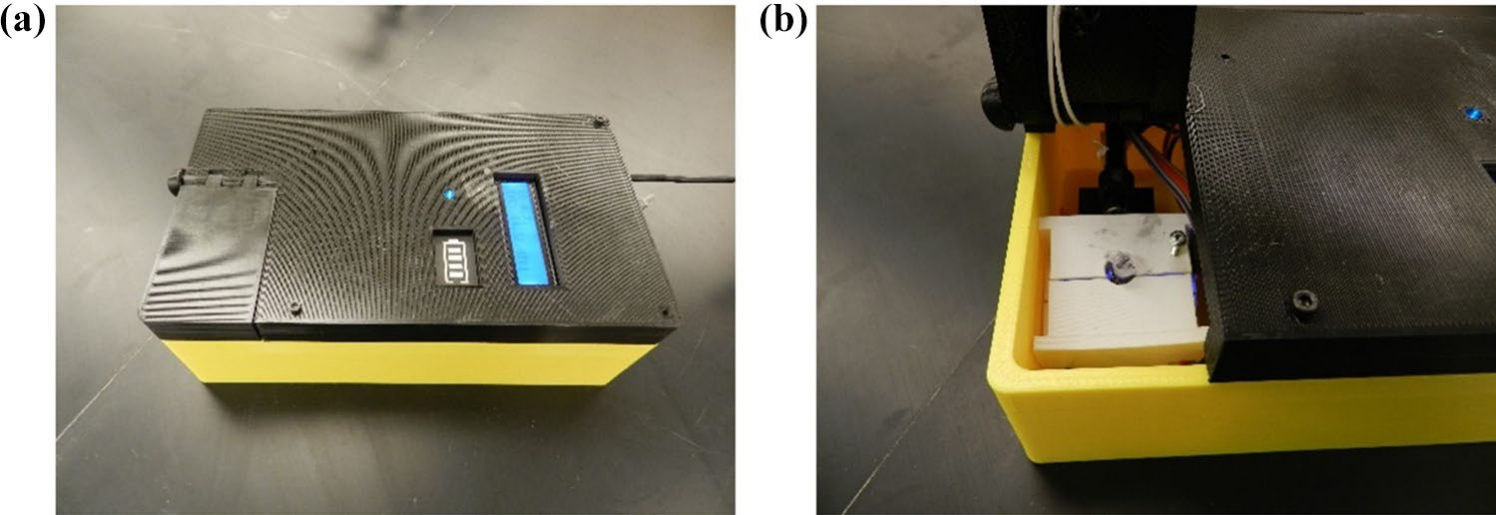
(**a**) 3d-printed apparatus for LAMP reaction, (**b**) cuvette holder heated by Peltier Module.

**Figure 3:**
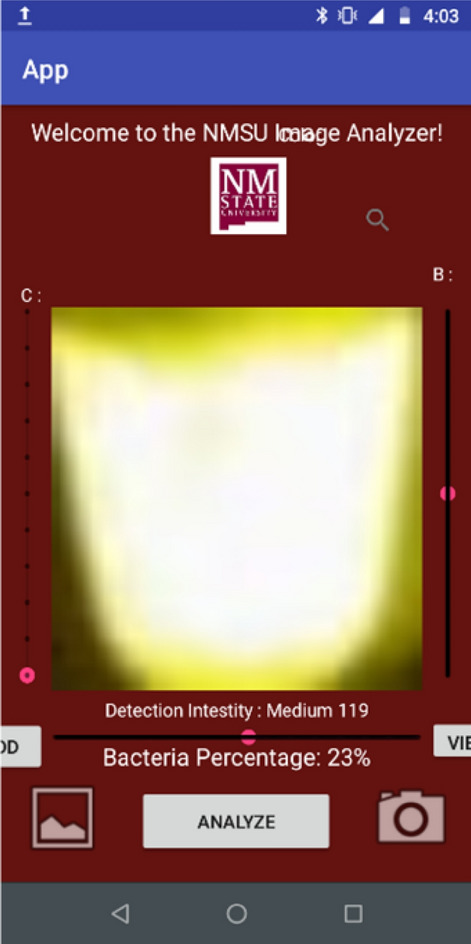
Fluorescent image of a sample captured by a smart phone application.

We developed a novel rapid detection platform that combines a user-friendly and portable LAMP reaction device (Fig. 1a) and the smartphone-based detection system (Fig. 3), which enables point-of-cared (POC) testing and diagnostics. The LAMP reaction device requires a heating element to heat up the fluorescence-based LAMP assay sample in the cuvette up to 65–70 °C. The Peltier module is used as the heating element in this device (see Figs. 1b and 2b). The Peltier module is a compact semi-conductor based electronic element that acts as a heat pump.

The device contains a printed circuit board (PCB) powered by an Arduino Nano microcontroller that allows the heating unit to reach and maintain a specific temperature and the system to notify the completion of the LAMP reaction. Four AA 2500-mAh rechargeable batteries are used to power the heating unit and the LED screen that displays temperature, battery charge level and heating completion message. These batteries also power a light-emitting diode (LED) to illuminate the cuvette containing the fluorescence-based assay sample. Figure 2 shows the 3D-printed apparatus for LAMP reaction and the cuvette holder. The excitation signal for the fluorescent dye is provide by an LED. The light beam is passed through the cuvette where a portion of light is absorbed and the rest is emitted. The light emission is captured by a USB camera installed within the device. USB cameras have a short focal length and are compatible with Android. The captured images are uploaded and analyzed on a smartphone app to correlate the fluorescence intensity with *S*. *aureus* concentration.

### Smartphone application programming

A smartphone software application (app) was developed and used to process images to quantify the bacterial count. The app can be used intuitively, requiring minimal input from an untrained user. The app algorithm for fluorescent signal measurement involves capturing, storing and processing the image of the sample^23^. Color information from the image is extracted and matched with the Red, Green, Blue (RGB) values to assist the user in selecting the target color. The source code of the android app was created in the Android Studio Java language. Once an image is captured by the app, the pixel analysis is processed and the percentage value of matching pixels throughout the entire image, which is correlated with the target nucleic acid concentration, is calculated and displayed by pressing the analyze button (Fig. 3). The main function of the application is to allow the user to define the color of the emitted fluorescent light that will be utilized to detect the target within the sample. Once an image is taken, the application allows the user to interact with the selected image and allows for the touched pixel of the image to determine the RGB value. Further details are found in Reference^23^.

## Results

### DNA extraction and LAMP amplification

**Figure 4:**
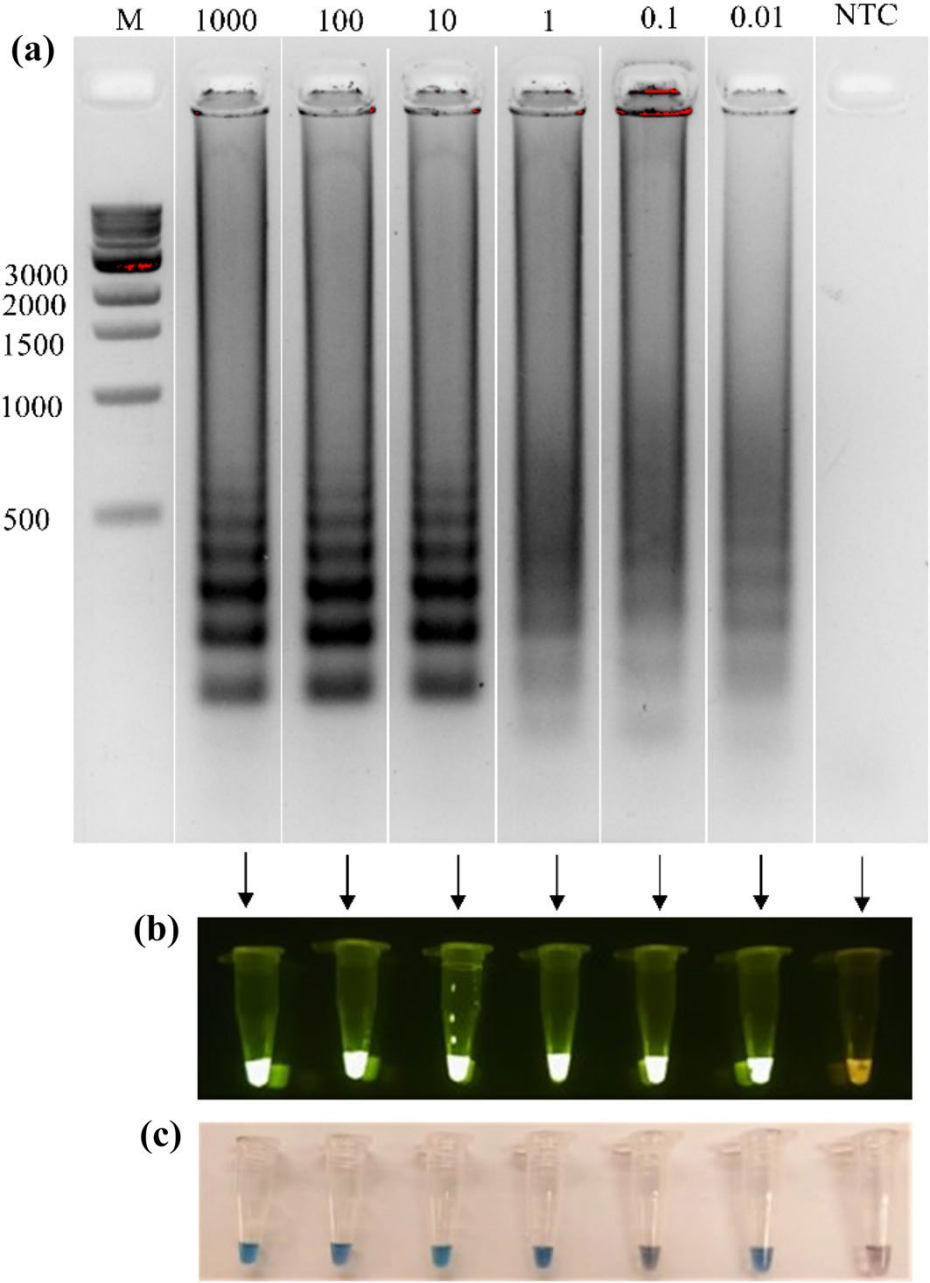
Sensitivity of LAMP amplification. (**a**) LAMP products visualized in 2% agarose gel containing different genomic DNA concentrations from *S. aureus* ranging from 1000 to 0.01 ng. DNA size marker is shown in leftmost lane; and a no template control (NTC) is shown in rightmost lane. “M” indicates “molecular-weight size marker.” (**b**) LAMP products with SYBR safe dye observed under blue light at different DNA concentrations. (**c**) LAMP products observed under naked eye with HNB dye with different DNA concentrations.

**Figure 5:**
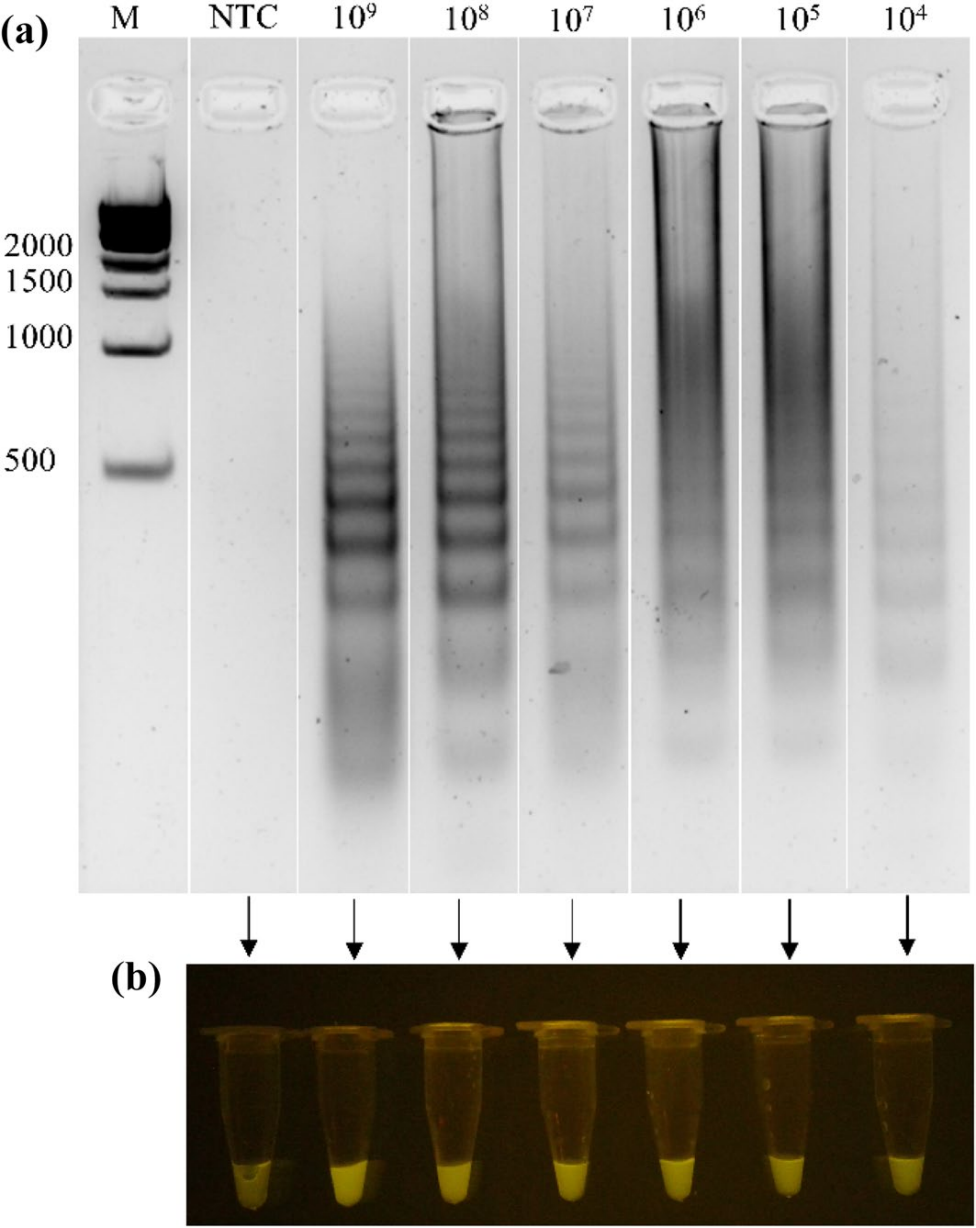
Sensitivity of LAMP in milk samples. (**a**) LAMP products visualized in 2% agarose gel. DNA size marker is shown in leftmost lane; and a no template control (NTC) is shown in rightmost lane. “M” indicates “molecular-weight size marker.” (**b**) LAMP amplification products with SYBR safe as a dye observed under blue light from milk samples contaminated at several concentrations of *S. aureus* (10^9^–10^4^ CFU/mL).

The quantification done with a nanodrop of genomic DNA from the *S. aureus* extraction yielded a concentration of 1480 ng/μL with a 230/260 ratio of 1.82. The visualization of the DNA in the agarose gel shows that the samples are not degraded. To evaluate the sensitivity of LAMP, different DNA concentrations were tested, ranging from 1000 ng to 10 pg. All the concentrations show the multiple bands, which is expected under this technique through agarose gel (Fig. 4a). With the SYBR Safe dye, the green–yellow fluorescence was observed under blue light on all samples containing the DNA template in comparison with the no template control (NTC) which had an orange–yellow color (Fig. 4b). On the other hand, reactions with HNB dye show a blue color in the positives and a purple color in the negative reaction (Fig. 4c). Sensitivity on direct detection of *S. aureus* in inoculated milk samples was observed from 10^9^ to 10^4^ CFU/mL (Fig. 5).

**Figure 6:**
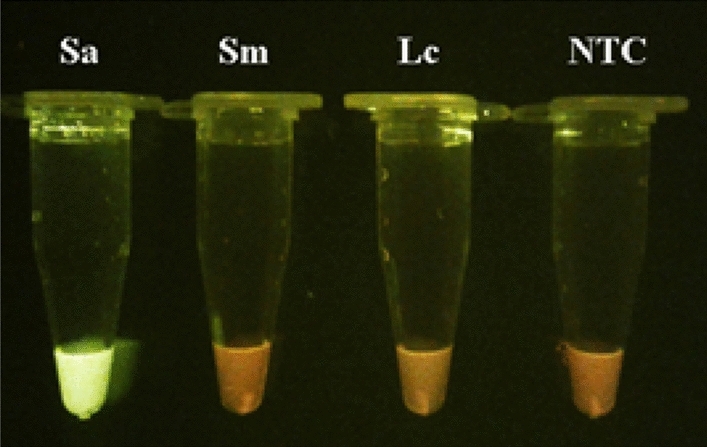
Specificity of LAMP primers to detect *S. aureus*. A LAMP reaction was developed against other species bacteria to evaluate their specificity against *S. aureus* (Sa). In both bacteria tested there is no green fluorescence indicating that there is no cross reaction with *Serratia marcescens* (*Sm*) and *Lactobacillus casei* (*Lc*); which show a coloration like the non-template tube (NTC).

To evaluate the specificity of the primers, DNAs from *Serratia marcescens* and *Lactobacillus casei* were employed as a control. Both strains show an orange color in the tube (Fig. 6), these assays prove that in-silico designed primers are targeting *S. aureus* and there is no cross detection with other bacterial strains.

### Mobile application for color sensitivity assay detection

**Figure 7:**
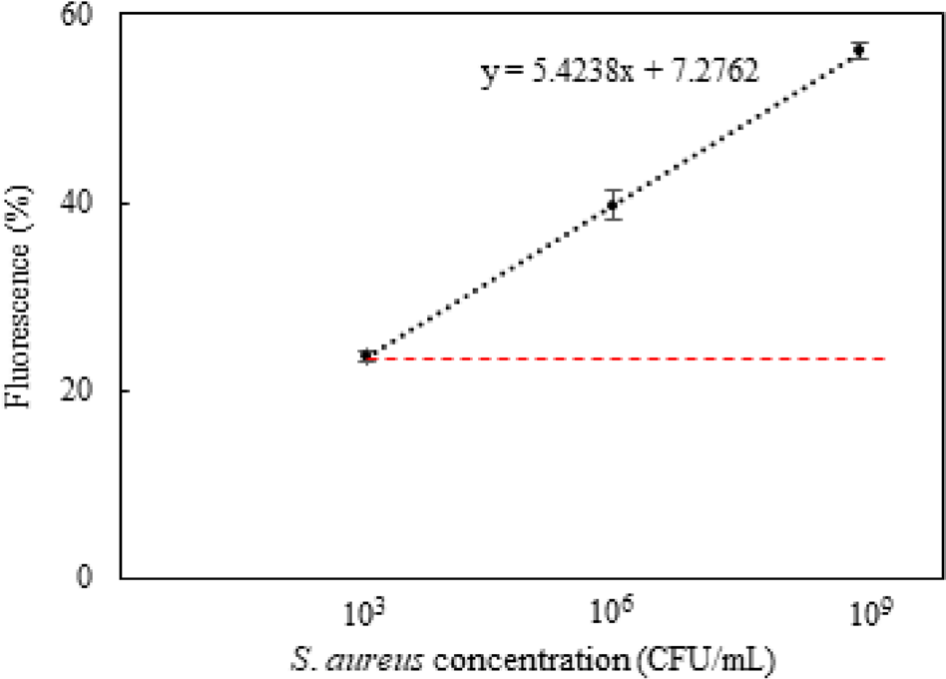
Fluorescence percentage (%) calculated by the smartphone app versus *S. aureus* concentration of LAMP assay with the cut-off (red dotted line).

Using the proposed smartphone detection system, we analyzed LAMP products with various bacterial load levels. Experiments were performed to measure LAMP assay samples containing different concentrations of *S*. *aureus* (10^9^ CFU/mL, 10^6^ CFU/mL, and 10^3^ CFU/mL). Our app showed testing results of quantifying *S. aureus* by the fluorescent dye. Figure 7 shows the average percentages of fluorescence detected in assay. The app accommodated samples with *S*. *aureus* concentration from 10^3^ to 10^9^ CFU/mL, and it is apparent that the percentage of fluorescence and concentration of *S*. *aureus* have a linear relationship. From the present proof-of-concept study, this platform offers a portable, easy-to-use method for measuring *S*. *aureus* concentration with LAMP amplification.

## Discussion

*Staphylococcus aureus* is one of the most important food-borne pathogen and control of *S. aureus* is a significant public health concern, especially in dairy products where several outbreaks of food poisoning have been identified^24^. *Staphylococcus aureus* can be introduced at almost every step of production. For this reason, it is crucial to implement food safety strategies to avoid contamination in handling and processing, as well as monitoring and diagnosing all these steps in a timely fashion to guarantee a pathogen-free product.

One of the most important concerns about *S. aureus* is the production of a wide array of toxins that can lead to food poisoning; however, staphylococcal food poisoning takes place when the pathogen populations exceed 10^5^ cells per gram^25^. In this work, we were able to detect a lower number of cells, which allows us to identify *S. aureus* accurately and rapidly before it reaches toxic levels. Some of the desired elements in a diagnostic technique are its sensitivity and speed to obtain the results. Since the LAMP technique was developed, it has been used as an important diagnostic tool. The LAMP is relatively easy to perform with high sensitivity. In this work we were not able to detect DNA concentrations lower than 10 pg using this set of primers. However, in the previous work of Sheet et al.^26^, *S. aureus* was detected by LAMP using primers based on the gene *nuc* with a sensitivity of 0.052 pg and 9 × 10^2^ CFU/mL. The femA gene was selected because of its high conservation in *S. aureus* isolates and its previous successful use as a molecular marker in PCR and recently in LAMP^27,28^.

In this work, the LAMP assay works with 0.01 ng of DNA. Previous study reports that 1 ng of DNA template or less is essential for the LAMP detection compared with the PCR detection where 10 ng are necessary. It makes LAMP 10 times more sensitive than PCR. Nevertheless, LAMP is less sensitive than nested PCR and qPCR^29^. To compare the sensitivity of LAMP versus qPCR for detecting *S. aureus*, we reproduced the assay reported by Kim et al.^30^ with genomic DNA from *S. aureus*. We were able to detect 1 pg of DNA and calculated a limit of detection (LOD) of 1.3 pg with qPCR. The result can be found in the supplementary information (Supplementary Fig. S1). This sensitivity analysis showed that qPCR was more sensitive than LAMP at detecting *S. aureus* DNA and CFU as a template, which is consistent with published results^30^. However, in terms of reaction time, LAMP reactions are very fast (< 60 min) as no thermocycling steps and specialized equipment are necessary; and results can be obtained in as little as 15 min^31^.

A common problem found in LAMP experiments is the amplification in the negative control; this issue has been reported in previous research. Also, the mispriming in LAMP reactions can result in incorrect interpretations such as false positives^32^. The provider recommends carrying out the standardization of the LAMP reaction. This can be done through the standardization of the pH, purity and ratio of the oligonucleotides, dye and DNA concentration as well as temperature and reaction time. Furthermore, cross contamination can occur when the lids of the reaction tube are opened. To prevent it, the use of agar capsules has been proposed^33^. In addition, precautions must be taken during the master mix preparation. For instance, working in separate areas and the use of micropipettes exclusive to the LAMP experiments, filter tips, DNAse and RNAse-free tubes and keeping the workstation clean through the use of bleach are recommended (Supplementary Figs. S2 and S3).

This project's LAMP reaction and detection platform offers closed-tube fluorescent monitoring that does not require opening the tube after amplification. The application developed and used in this study calculated the percentage value of matching pixels throughout the entire image using the selected RGB value of the fluorescent signal. This percentage value was correlated with the bacterial load concentration for which the coefficient of determination (R^2^) equals 0.986. This study combines an effective image processing algorithm coded into a smartphone app with an inexpensive 3D printed apparatus that eliminated inaccurate visual monitoring and allowed for the quantification of bacterial load and nucleic acid concentration. The portable LAMP reaction and detection platform will eliminate the need for dedicated laboratory equipment for thermal cycling to operate polymerase chain reaction (PCR) amplification. The present closed tube amplification eliminates the risk of carry-over contamination and enables the real-time quantification of the amplified DNA for endpoint real-time analysis. From the present proof-of-concept study, this platform based on fluorescence imaging/detection offers a portable, simplified method to measure target pathogens with LAMP amplification.

## Supplementary Information

Supplementary Figure S1.

Supplementary Figure S2.

Supplementary Figure S3.

## Data Availability

The data sets used in this study are available upon reasonable request from the corresponding authors.
