# Supplementary material for: Rapid identification of Staphylococcus aureus based on a fluorescence imaging/detection platform that combines loop mediated isothermal amplification assay and the smartphone-based system
Source: Sci Rep. 2022 Nov 30;12:20655. doi: 10.1038/s41598-022-25190-6 (PMC9712598; doi:10.1038/s41598-022-25190-6)

Supplementary Figure 1. Detection of *S. aureus* by qPCR. Genomic DNA of *S. aureus* at concentrations from 100 ng to 1 pgwere detected by qPCR. Samples were analyzed by duplicate. LOD = [3.3*(σ/s)]


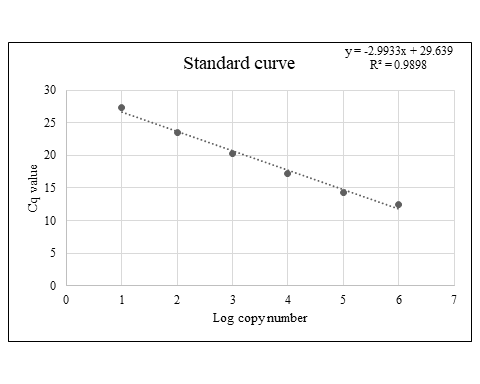

Supplement: Supplementary file 1 — Supplementary Figure S1. [file 41598_2022_25190_MOESM1_ESM.docx]
