# Supplementary material for: Rapid identification of Staphylococcus aureus based on a fluorescence imaging/detection platform that combines loop mediated isothermal amplification assay and the smartphone-based system
Source: Sci Rep. 2022 Nov 30;12:20655. doi: 10.1038/s41598-022-25190-6 (PMC9712598; doi:10.1038/s41598-022-25190-6)

Supplementary Figure 2. The original image of gel electrophoresis of *S* *aureus*-specific LAMP reaction ranging from 1000 ng to 0.01 ng


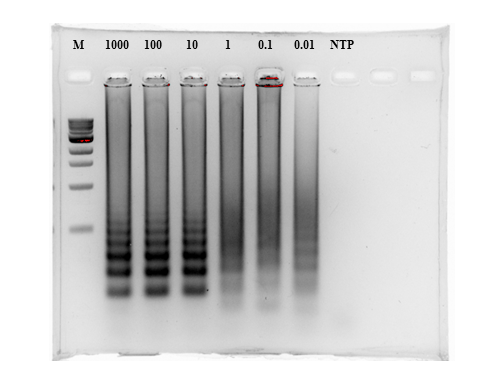

Supplement: Supplementary file 2 — Supplementary Figure S2. [file 41598_2022_25190_MOESM2_ESM.docx]
