# Supplementary material for: Rapid identification of Staphylococcus aureus based on a fluorescence imaging/detection platform that combines loop mediated isothermal amplification assay and the smartphone-based system
Source: Sci Rep. 2022 Nov 30;12:20655. doi: 10.1038/s41598-022-25190-6 (PMC9712598; doi:10.1038/s41598-022-25190-6)

Supplementary Figure 3. The original image of gel electrophoresis of *S* *aureus* in inoculated milk sample


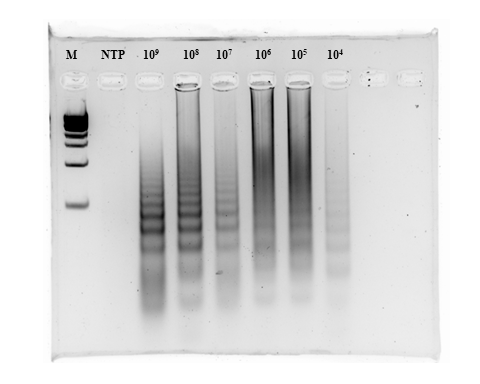

Supplement: Supplementary file 3 — Supplementary Figure S3. [file 41598_2022_25190_MOESM3_ESM.docx]
